# Supplementary material for: High hydrostatic pressure stimulates microbial nitrate reduction in hadal trench sediments under oxic conditions
Source: Nat Commun. 2024 Mar 19;15:2473. doi: 10.1038/s41467-024-46897-2 (PMC10951307; doi:10.1038/s41467-024-46897-2)
Supplement: Supplementary file 1 — Supplementary Information [file 41467_2024_46897_MOESM1_ESM.pdf]

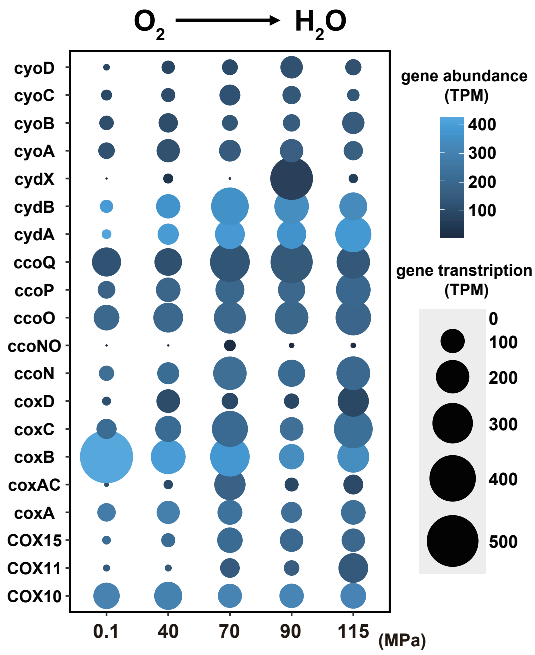

Supplementary Fig. 1 The different colors represent the abundance (TPM values) and the point size shows the transcripts (TPM values) of the aerobic respiration genes under different hydrostatic pressures.

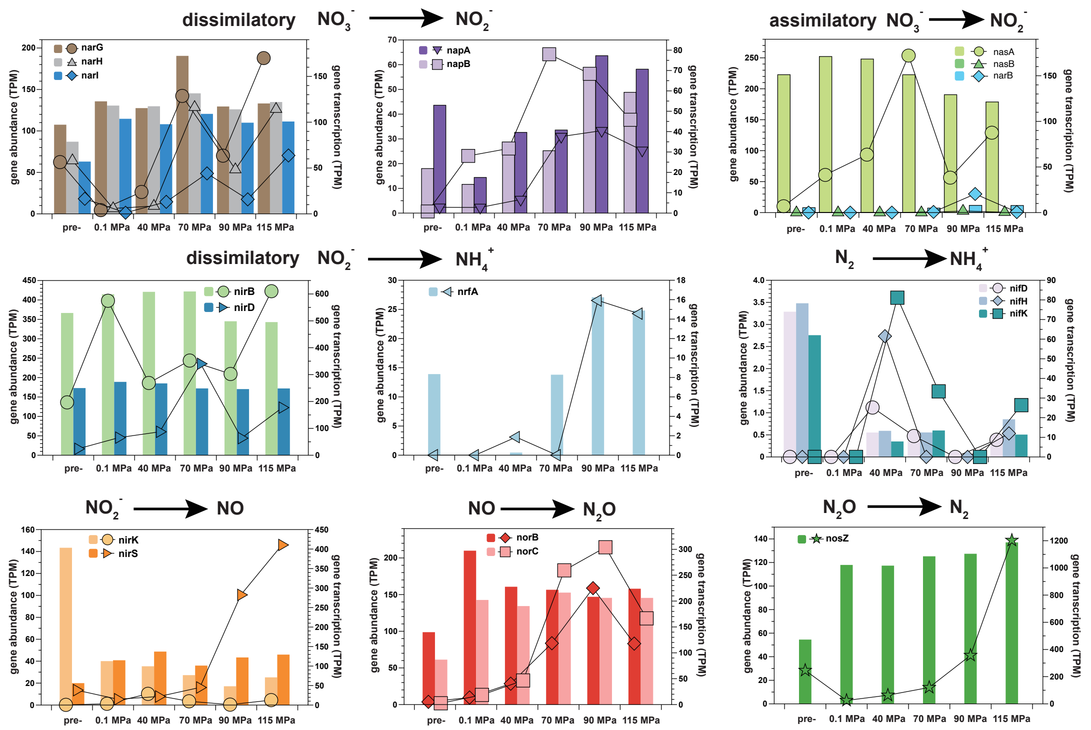

Supplementary Fig. 2 Bar plots showing the abundance (TPM values) of nitrogen-cycling genes under different hydrostatic pressures. Dot-line plots showing the transcript (TPM values) of nitrogen-cycling genes under different hydrostatic pressures.

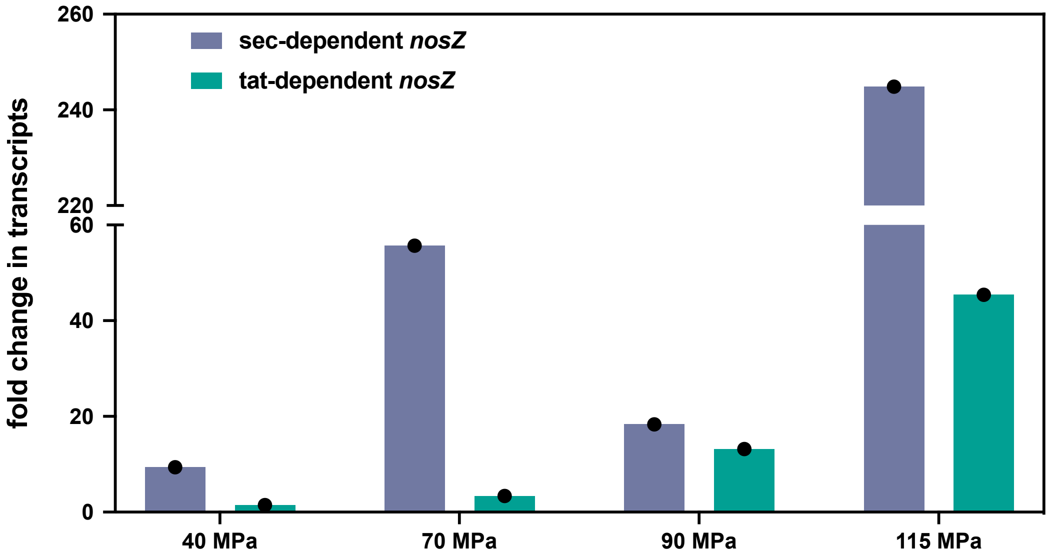

Supplementary Fig. 3 The increase fold versus 0.1 MPa (expressed as (TPM_x-$TPM_{0.1\ MPa}$)/$TPM_{0.1\ MPa}$) in gene transcripts (TPM) of sec-dependent *nosZ* and tat-dependent *nosZ* genes with elevated hydrostatic pressures. Note: since the sec-dependent *nosZ* gene transcript was 0 at 0.1 MPa, to compare the increase fold relative to 0.1 MPa, the transcript value was artificially set to 0.2.

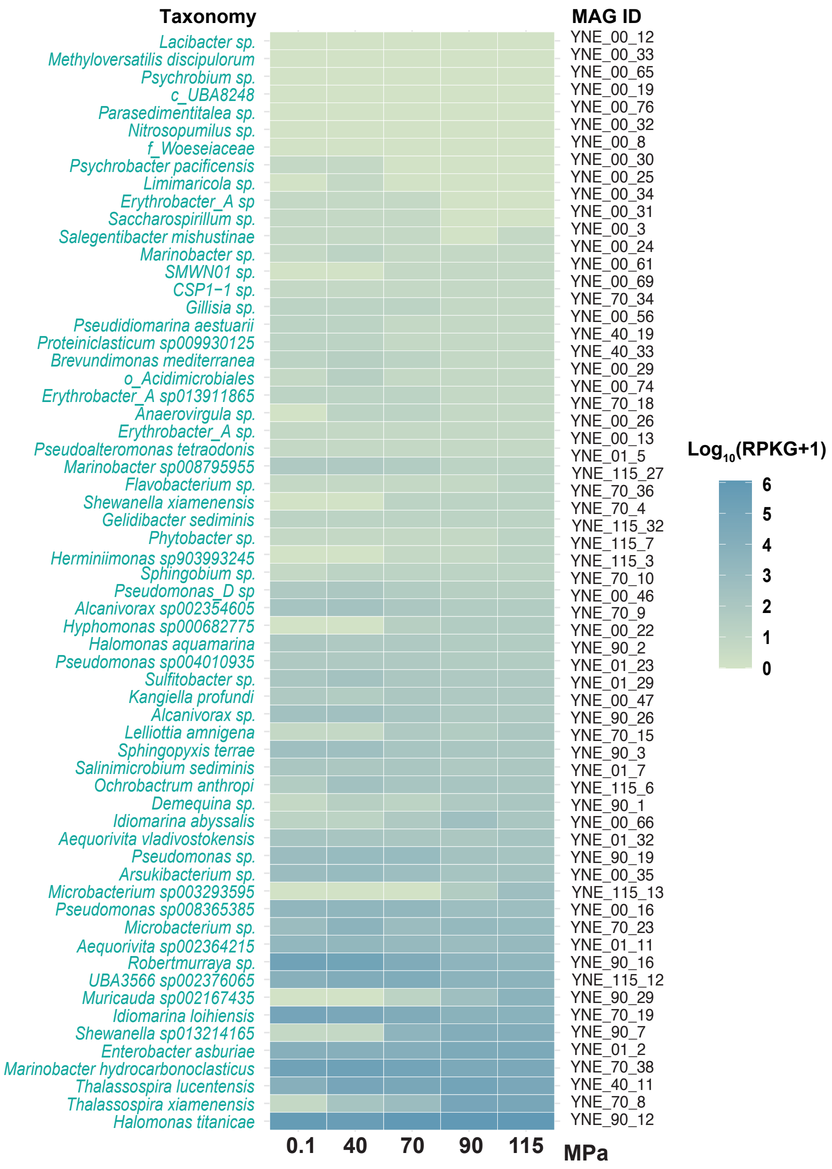

Supplementary Fig. 4 The normalized abundance (RPKG) of the recovered MAGs from five metagenomic datasets derived from flow-incubation sediment samples.

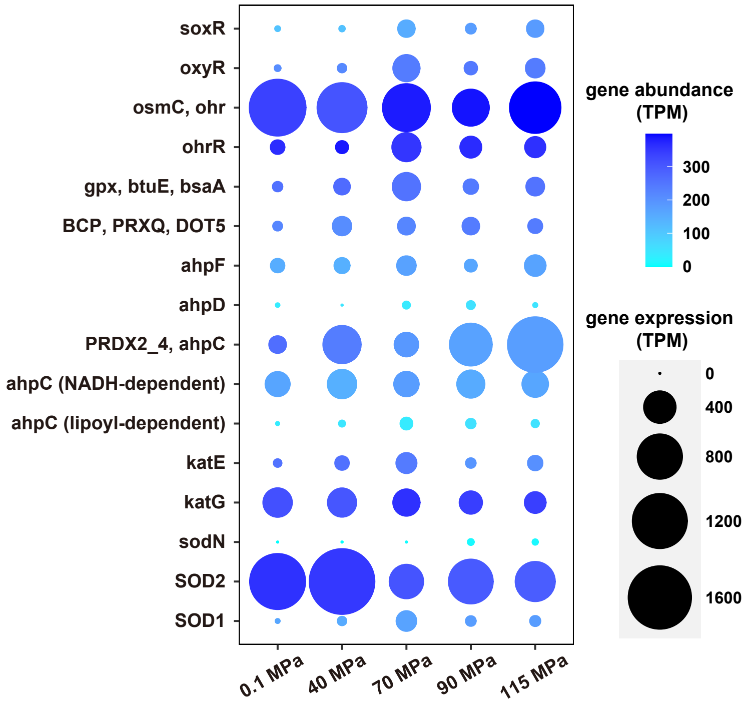

Supplementary Fig. 5 Abundance of reactive oxygen species (ROS) scavenging genes. The color represents the abundance in metagenomic datasets and the size represents the abundance in metatranscriptomic datasets.
