# Supplementary material for: High hydrostatic pressure stimulates microbial nitrate reduction in hadal trench sediments under oxic conditions
Source: Nat Commun. 2024 Mar 19;15:2473. doi: 10.1038/s41467-024-46897-2 (PMC10951307; doi:10.1038/s41467-024-46897-2)
Supplement: Supplementary file 3 — Description of Additional Supplementary Files [file 41467_2024_46897_MOESM3_ESM.pdf]

Description of Additional Supplementary Files

Supplementary Data 1. Biomass (cell numbers) in the inoculum sediment and continuous flowing incubation sediment samples under different hydrostatic pressures.

Supplementary Data 2. The relative abundance of bacterial and archaeal community composition at family level in continuous flowing incubation sediment samples under different hydrostatic pressures.

Supplementary Data 3. The microbial net nitrogen metabolism activity over time under each hydrostatic pressure and mean values of microbial net nitrogen metabolism activity were calculated under each hydrostatic pressure.

Supplementary Data 4. The gene abundance (TPM) and gene transcripts (TPM) involved in nitrogen-cycling pathways in the inoculum sediment and continuous flowing incubation sediment samples under different hydrostatic pressures.

Supplementary Data 5. The detailed information of abundance (TPM) and transcript (TPM) for the Sec-dependent *nosZ* and Tat-dependent *nosZ* gene clusters under different hydrostatic pressures.

Supplementary Data 6.
a. The N_2O consumption by Tat-dependent and Sec-dependent *nosZ* genes after incubation at 0.1, 20 and 40 MPa for 24 h.
b. The transcriptional activity (TPM) of Tat-dependent and Sec-dependent *nosZ* genes after incubation at 0.1, 20 and 40 MPa for 24 h.

Supplementary Data 7. The detailed information of all MAGs retrieved from the inoculum sediment and continuous flowing incubation sediment samples under different hydrostatic pressures in this study.

Supplementary Data 8. The retrieved MAGs that actively involved in denitrification pathways in the inoculum sediment and continuous flowing incubation sediment samples under different hydrostatic pressures. These MAGs ID in red font indicate that denitrification-related genes in this MAG are transcribed actively under at least one hydrostatic pressure condition. For *nosZ* genes, the green font indicates Tat-dependent *nosZ* genes present in this MAG, and the purple font indicates Sec-dependent *nosZ* genes present in this MAG.

Supplementary Data 9. The MAGs involved in denitrification and/or aerobic respiration pathways in the inoculum sediment and continuous flowing incubation sediment samples under different hydrostatic pressures. A yellow background indicates that both aerobic respiration-related genes and denitrification-related genes in this MAG are transcribed actively under at least one hydrostatic pressure condition. A gray background indicates that although these two pathway-related genes are present in this MAG, they are not transcribed simultaneously. A white background indicates the presence of aerobic respiration-related genes in the MAG, but no denitrification-related genes.

Supplementary Data 10. The transcripts (TPM) via metatranscriptomes of genes involved in denitrification and aerobic respiration pathways in sediment from the Mariana Trench.
