# Supplementary material for: High hydrostatic pressure stimulates microbial nitrate reduction in hadal trench sediments under oxic conditions
Source: Nat Commun. 2024 Mar 19;15:2473. doi: 10.1038/s41467-024-46897-2 (PMC10951307; doi:10.1038/s41467-024-46897-2)
Supplement: Supplementary file 5 — Reporting Summary [file 41467_2024_46897_MOESM5_ESM.pdf]

nature portfolio

Corresponding author(s): Yu Zhang

Last updated by author(s): Mar 4, 2024

Reporting Summary

Nature Portfolio wishes to improve the reproducibility of the work that we publish. This form provides structure for consistency and transparency in reporting. For further information on Nature Portfolio policies, see our Editorial Policies and the Editorial Policy Checklist.

Statistics

For all statistical analyses, confirm that the following items are present in the figure legend, table legend, main text, or Methods section.

n/a	Confirmed	
☐	☒	The exact sample size (*n*) for each experimental group/condition, given as a discrete number and unit of measurement
☐	☒	A statement on whether measurements were taken from distinct samples or whether the same sample was measured repeatedly
☒	☐	The statistical test(s) used AND whether they are one- or two-sided<br>*Only common tests should be described solely by name; describe more complex techniques in the Methods section.*
☒	☐	A description of all covariates tested
☒	☐	A description of any assumptions or corrections, such as tests of normality and adjustment for multiple comparisons
☐	☒	A full description of the statistical parameters including central tendency (e.g. means) or other basic estimates (e.g. regression coefficient) AND variation (e.g. standard deviation) or associated estimates of uncertainty (e.g. confidence intervals)
☐	☒	For null hypothesis testing, the test statistic (e.g. *F*, *t*, *r*) with confidence intervals, effect sizes, degrees of freedom and *P* value noted<br>*Give P values as exact values whenever suitable.*
☒	☐	For Bayesian analysis, information on the choice of priors and Markov chain Monte Carlo settings
☒	☐	For hierarchical and complex designs, identification of the appropriate level for tests and full reporting of outcomes
☒	☐	Estimates of effect sizes (e.g. Cohen's *d*, Pearson's *r*), indicating how they were calculated

Our web collection on statistics for biologists contains articles on many of the points above.

Software and code

Policy information about availability of computer code

Data collection	The concentrations of dissolved inorganic nitrogen species (NO3-, NO2-, and NH4+) were quantified using an AA3 AutoAnalyzer system (Seal Analytical, UK). The concentration of gas N2O was measured by an Agilent 6890N Gas Chromatograph (Agilent Technologies, USA). The initial concentration of dissolved oxygen was measured using the Microx 4 oxygen meter (PreSens, Germany). The quantity and quality of extracted total DNA and RNA of sediments were measured using Qubit 4.0 Fluorometer (Invitrogen, Carlsbad, CA, USA). The transcriptomic, metagenomic and metatranscriptomic libraries were sequenced by Illumina NovaSeq 6000 platform (Illumina, USA). The 16S rRNA gene amplicon was sequenced on the Illumina NovaSeq 6000 platform (Illumina, USA). The 7500 Real-Time PCR System (Applied Biosystems, Foster City, CA, USA) was used for all qPCR analysis.
Data analysis	QIIME2 (v.2020.8); BBDuk tool (v.38.96); SPAdes (v.3.12.0); Prodigal (v.2.6.3); GhostKOALA (v.2.2); emapper (v.2.0.1) against the EggNOG database (v.5.0); KofamKOALA (v.1.0.3); BBMap (v.38.24); SAMtools (v.1.15.1); Bowtie2 (v.2.4.1); MetaBAT2 (v.2.15); MaxBin (v.2.2.6); FeatureCounts (v.1.5.3); CONCOCT (v.1.0.0); DAStools (v.1.2.2); GTDB-Tk tools (v.2.3.2); CheckM (v.1.1.5); dRep (v.3.4.2); IQ-TREE tool (v.2.1.2)

For manuscripts utilizing custom algorithms or software that are central to the research but not yet described in published literature, software must be made available to editors and reviewers. We strongly encourage code deposition in a community repository (e.g. GitHub). See the Nature Portfolio guidelines for submitting code & software for further information.

Data

Policy information about availability of data

All manuscripts must include a data availability statement. This statement should provide the following information, where applicable:

- Accession codes, unique identifiers, or web links for publicly available datasets
- A description of any restrictions on data availability
- For clinical datasets or third party data, please ensure that the statement adheres to our policy

The raw 16S rRNA gene amplicon reads, metagenomic and metatranscriptomic data of flowing incubation samples, the transcriptomic data of recombinant WP3NR strains, and the metatranscriptomic data of in situ fixed sediments in the Mariana Trench generated in this study have been deposited to the National Omics Data Encyclopedia (NODE, https://www.biosino.org/node/index) database under the accession numbers OEP004015, OEP004042, OEP004045, OEP004102, OEP004512, as well as the NCBI SRA database under the BioProject IDs of PRJNA1083314, PRJNA1083644, PRJNA1083643, PRJNA1083642. The databases used in this study include SILVA 138 database (https://www.arb-silva.de/documentation/release-138/), GTDB database Release 202 (https://data.gtdb.ecogenomic.org/releases/release202/), and KEGG database (https://www.genome.ad.jp/kegg/). All other data are available in this paper or the Supplementary Information. Source data are provided with this paper.

Research involving human participants, their data, or biological material

Policy information about studies with human participants or human data. See also policy information about sex, gender (identity/presentation), and sexual orientation and race, ethnicity and racism.

Reporting on sex and gender	N/A
Reporting on race, ethnicity, or other socially relevant groupings	N/A
Population characteristics	N/A
Recruitment	N/A
Ethics oversight	N/A

Note that full information on the approval of the study protocol must also be provided in the manuscript.

Field-specific reporting

Please select the one below that is the best fit for your research. If you are not sure, read the appropriate sections before making your selection.

☐ Life sciences ☐ Behavioural & social sciences ☒ Ecological, evolutionary & environmental sciences

For a reference copy of the document with all sections, see nature.com/documents/nr-reporting-summary-flat.pdf

Ecological, evolutionary & environmental sciences study design

All studies must disclose on these points even when the disclosure is negative.

Study description	Hadal trenches represent one of Earth's most extreme environments. They are situated over 6000 m below the abyssal realm, where the enormous hydrostatic pressure affects the biochemical cycling of elements. Recent studies have indicated that hadal trenches may represent a previously overlooked source of fixed nitrogen loss, however, the mechanisms and the role of hydrostatic pressure in this process are still being debated. To this end, we investigated the effects of hydrostatic pressure (ranging from 0.1 to 115 MPa) on the chemical profile, microbial community structure, and functions of surface sediments from the Mariana Trench using a Deep Ocean Experimental Simulator supplied with nitrate and oxygen. We observed enhanced denitrification activity at high hydrostatic pressure under oxic conditions, while the anaerobic ammonium oxidation (anammox) – a previously recognized dominant nitrogen loss pathway - was not detected. Additionally, we further confirmed the simultaneous occurrence of nitrate reduction and aerobic respiration using a metatranscriptomic dataset from in situ RNA-fixed sediments in the Mariana Trench. Taken together, our findings demonstrate that hydrostatic pressure can restructure the chemical zonation in hadal trenches and the hadal trenches are a potential nitrogen loss hotspot. Knowledge of the influence of hydrostatic pressure on anaerobic processes in oxygenated surface sediments could greatly broaden our understanding of the biochemical cycling of key elements in hadal trenches.
Research sample	The surface sediment samples were collected in the Mariana Trench (142.2516 °E, 11.6639 °N) at water depth of 6002 m in June 2016, which were used for incubation experiments. Additional sediment samples were collected in the Mariana Trench at water depth of 8207–10898 m from August to December 2021. These samples were fixed with RNALater immediately after acquisition, which were used for metatranscriptomic analysis.
Sampling strategy	The surface sediment samples used in the flowing-incubation experiments were obtained by push cores operated by the HOV Jiaolong in the Mariana Trench. Additional sediment samples used for metatranscriptomic analysis to describe the in situ microbial

community were collected by an in-situ sediment-fixed sampler operated by HOV Fendouzhe in the Mariana Trench. For flowingincubation experiments, the consumption of nitrate, as well as the production of ammonium, nitrite, and nitrous oxide were analyzed to monitor the metabolic activities. Sampling of the above chemical parameters was conducted every 3 days without depressurization throughout the incubation experiments. Biomass sampling was performed when switching incubation pressure (ranging from 0.1 to 115 MPa). For the activity assay of Tat-dependent N2O reductase and Sec-dependent N2O reductase under 0.1, 20 and 40 MPa, sampling was taken at 0 h and 24 h to detect N2O consumption and transcriptional activity, respectively.

Data collection

For each sediment samples, Na Yang extracted the total DNA using the modified SDS-based method and extracted the total RNA using the RNeasy® PowerSoil® Total RNA Kit (Qiagen, Germany) according to the manufacturer's instructions. Following extraction, total nucleic acids were sequenced on Illumina NovaSeq 6000 platform (Illumina, USA) with PE150 strategy at Shanghai Personal Biotechnology. Following sequencing, Na Yang and Yongxin Lv analyzed the metagenomic and metatranscriptomic data.
Na Yang performed the 16S rRNA gene PCR amplification of the extracted DNA from each sediment sample using the primer pairs Arch516F/Arch855R and Bac533F/Bac806R. Na Yang performed the quantitative PCR analysis of the extracted DNA from each sediment sample using 7500 Real-Time PCR System and PowerUp SYBR Green Master Mix (2X) (Applied Biosystems, Foster City, CA, USA).
Na Yang measured all chemical profiles (dissolved inorganic nitrogen species, including NO3-, NO2- and NH4+; gaseous nitrogen, including N2O) using an AA3 AutoAnalyzer system and Agilent 6890N Gas Chromatograph, respectively.
Na Yang performed the heterologous expression of Tat-dependent N2O reductase and Sec-dependent N2O reductase in deep-sea model bacterium Shewanella piezotolerans WP3NR. Na Yang performed the activity assay of Tat-dependent N2O reductase and Secdependent N2O reductase by measuring N2O consumption. Na Yang extracted the RNA of three WP3NR transconjugants using common TRIzol method. Following extraction, RNA was sequenced on Illumina platform at Shanghai Personal Biotechnology. Following sequence, Na Yang performed the subsequent transcriptomic data analysis.
For each sediment samples, Na Yang extracted the total DNA using the modified SDS-based method and extracted the total RNA using the RNeasy® PowerSoil® Total RNA Kit (Qiagen, Germany) according to the manufacturer's instructions. Following extraction, total nucleic acids were sequenced on Illumina NovaSeq 6000 platform (Illumina, USA) with PE150 strategy at Shanghai Personal Biotechnology. Following sequencing, Na Yang and Yongxin Lv analyzed the metagenomic and metatranscriptomic data.
Na Yang performed the 16S rRNA gene PCR amplification of the extracted DNA from each sediment sample using the primer pairs Arch516F/Arch855R and Bac533F/Bac806R. Na Yang performed the quantitative PCR analysis of the extracted DNA from each sediment sample using 7500 Real-Time PCR System and PowerUp SYBR Green Master Mix (2X) (Applied Biosystems, Foster City, CA, USA).
Na Yang measured all chemical profiles (dissolved inorganic nitrogen species, including NO3-, NO2- and NH4+; gaseous nitrogen, including N2O) using an AA3 AutoAnalyzer system and Agilent 6890N Gas Chromatograph, respectively.
Na Yang performed the heterologous expression of Tat-dependent N2O reductase and Sec-dependent N2O reductase in deep-sea model bacterium Shewanella piezotolerans WP3NR. Na Yang performed the activity assay of Tat-dependent N2O reductase and Secdependent N2O reductase by measuring N2O consumption. Na Yang extracted the RNA of three WP3NR transconjugants using common TRIzol method. Following extraction, RNA was sequenced on Illumina platform at Shanghai Personal Biotechnology. Following sequence, Na Yang performed the subsequent transcriptomic data analysis.

Timing and spatial scale

The data collection started at May 2021 and ended at December 2022. The flow incubation experiment lasted 75 days. The flow incubation sediment samples were collected every 15 days for microbiology analysis, and chemical profiles were sampled every 3 days for biochemistry analysis before changing pressures (ranging from 0.1 to 115 MPa). After all the parameters in the study are tested, the data analysis process is carried out. The data obtained from laboratory incubation sample are not applicable to spatial scales.

Data exclusions

No data were excluded from the analysis.

Reproducibility

Heterologous expression and activity assay including transcriptomic sequencing have three biological replicates, quantitative PCR has three biological replicates and three technical replicates. All software versions and bioinformatics analysis are documented for reproducibility.

Randomization

All samples were analyzed; hence no randomization is necessary or possible.

Blinding

In this study, we collected data in our laboratory flow incubation experiments using pressure-retaining sediment samples and in situ RNA-fixed sediments in the Mariana Trench, so the blinding is not relevant to this study.

Did the study involve field work? ☒ Yes ☐ No

Field work, collection and transport

Field conditions

The initial sediment samples were obtained at Mariana Trench during DY37-II cruise in June 2016. The in situ fixed sediment samples were collected on Tansuoyihao cruise TS-21 from August to December 2021.

Location

The surface sediment samples were obtained from the northern slope of the Mariana Trench (142.2516°E, 11.6639°N). The in situ fixed sediments were collected from the Mariana Trench (142.5947 °E, 11.3649 °N; 142.5926 °E, 11.3867 °N; 142.5869 °E, 11.3740 ° N; 142.5602 °E, 11.3619 °N; 142.5602 °E, 11.3619 °N; 142.1562 °E, 11.1615 °N; 142.1572 °E, 11.1590 °N; 142.3429 °E, 11.1970 °N; 142.3429 °E, 11.1970 °N; 142.2038 °E, 11.3393 °N; 142.2166 °E, 11.3350 °N).

Access & import/export

Sample collection and transportation have been permitted by the Federated States of Micronesia. The permit number is FM-

	XXRS-23522. The date of approval is from 23 Aug to 21 Nov 2021.
Disturbance	No disturbance caused by this study.

Reporting for specific materials, systems and methods

We require information from authors about some types of materials, experimental systems and methods used in many studies. Here, indicate whether each material, system or method listed is relevant to your study. If you are not sure if a list item applies to your research, read the appropriate section before selecting a response.

Materials & experimental systems

n/a	Involved in the study
☒	☐ Antibodies
☒	☐ Eukaryotic cell lines
☒	☐ Palaeontology and archaeology
☒	☐ Animals and other organisms
☒	☐ Clinical data
☒	☐ Dual use research of concern
☒	☐ Plants

Methods

n/a	Involved in the study
☒	☐ ChIP-seq
☒	☐ Flow cytometry
☒	☐ MRI-based neuroimaging

Plants

Seed stocks	N/A
Novel plant genotypes	N/A
Authentication	N/A
